# Sulfated Polysaccharides Isolated from Cloned *Grateloupia filicina* and Their Anticoagulant Activity

**DOI:** 10.1155/2015/612352

**Published:** 2015-04-07

**Authors:** Xiaolin Chen, Shengfeng Yang, Jinxia Wang, Lin Song, Ronge Xing, Song Liu, Huahua Yu, Pengcheng Li

**Affiliations:** ^1^Institute of Oceanology, Chinese Academy of Sciences, No. 7 Nanhai Road, Qingdao, Shandong 266071, China; ^2^Qingdao Tumor Hospital, No. 127 Siliu Nan Road, Qingdao, Shandong 266042, China

## Abstract

Sulfated polysaccharides (GSP) were isolated from the cloned *Grateloupia filicina* which was cultured in Jiaozhou Bay, Qingdao, China. The yield of GSP was 15.75%. The total sugar and sulfate were 40.90 and 19.89%, respectively. And the average molecular weight was 11.7 KDa. The results of neutral sugar analysis showed that GSP was mainly sulfated polysaccharides of galactose. The experiments for activated partial thromboplastin time (APTT), prothrombin time (PT), and thrombin time (TT) anticoagulant assays in vitro indicated that GSP was a good potential anticoagulant. Therefore, this study supplied new thought for the cloned *Grateloupia filicina* exploitation of high-value products.

## 1. Introduction

Sulfated polysaccharides are widespread in nature. They have been found in animals, plants, and microorganisms. The majority of plant polysaccharides are present in algae (fucans, fucoidans, carrageenans, etc.). Sulfated polysaccharides from seaweeds have been widely studied for their chemical properties and biological activities in food and medical industries [1–3]. Among these activities, anticoagulant properties were studied more and more recently. Researchers have found and isolated various sulfated polysaccharides from brown algae, green algae, and red algae, which were reported to have anticoagulant properties [4–6].

On the other hand, according to World Health Organization, cardiovascular diseases including heart diseases and stroke related to thrombosis are the main cause of death globally and predictions have been made that, by 2030, almost 3.6 million people will die from these diseases [7]. Heparin as a sulfated polysaccharide has been used as an anticoagulant drug in the area of hematology and transfusion medicine for more than 50 years. However, it has several disadvantages such as production difficulties, chemical inhomogenicity, variability in physiological activities, and bleeding [8, 9]. Hence it is necessary to find safe, natural, and easy to use drug instead of heparin. Sulfated polysaccharides from marine seaweeds share similar ionic structure with heparin, so the sulfated polysaccharides have shown the anticoagulant activity. Therefore, sulfated polysaccharides may become a substitute of heparin.

**Figure 1:**
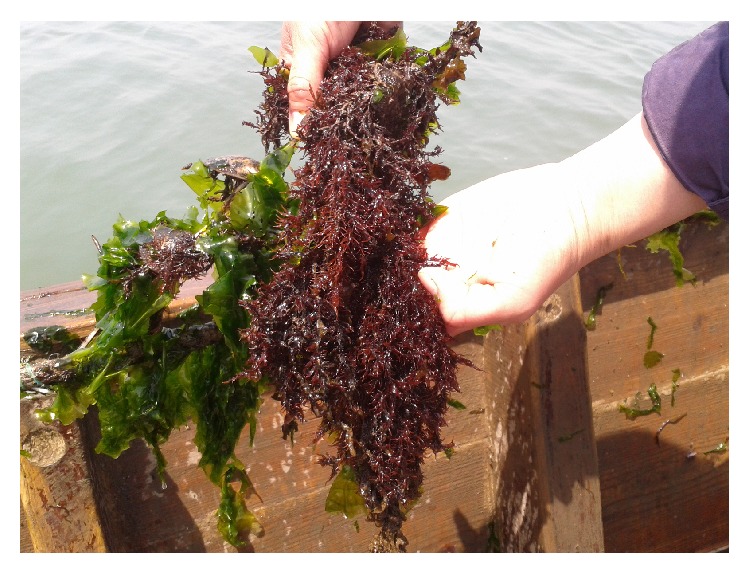
The cultivated* Grateloupia filicina* in Jiaozhou Bay, Qingdao, China.

Red alga* Grateloupia filicina* belongs to Rhodophyceae and grows in the Pacific, Indian, and warmer parts of the Atlantic oceans. This species is used as a food and as a source of carrageenan in the western Pacific [10, 11]. For red algae,* Grateloupia filicina* is rarely reported regarding its anticoagulant activity mainly because it is mostly wild and the obtained seaweed is limited. In our lab, we have cloned* Grateloupia filicina* by the totipotency of marine algae [12]. And the macroalgae have been cultivated in Jiaozhou Bay, Qingdao, China.  Figure 1 was the cultivated* Grateloupia filicina*.

In this paper, the sulfated polysaccharides with high molecular weight were extracted from the above cultivated* Grateloupia filicina *by hot water. The chemical composition and structure were analyzed. And the anticoagulant activity of the obtained polysaccharides was determined in the coagulation assays prothrombin time (PT), activated partial thromboplastin time (APTT), heptest, and thrombin time (TT) which record influences on different steps of the coagulation cascade. And the relationship between chemical properties and anticoagulant activity was described. This research can help to elucidate suitable and accessible anticoagulant drugs and utilize the red algae better.

## 2. Experimental

### 2.1. Materials

*Grateloupia filicina* was collected on Jiaozhou Bay in May 2013. And it was washed and dried at 60°C in oven. Then it was milled and kept in plastic bags at room temperature. Dialysis membranes (flat width 44 mm, molecular weight cut-off 3500) were purchased from Qingdao Qunheng Biological Technology Co., Ltd. Standard D-glucose, L-rhamnose, D-xylose, L-arabinose, D-mannose, L-fucose, D-galactose, and D-glucuronic acid were purchased from Sigma (St. Louis, Missouri, USA). Reagents for activated partial thromboplastin time (APTT), prothrombin time (PT), and thrombin time (TT) were purchased from Shang Hai Sun Biotechnology Co. Ltd. All other reagents were of analytical grade.

Blood sample was supplied by a normal adult male volunteer with type B blood. The volunteer was informed about the benefits and possible risks of the study. And signed informed consent was subsequently obtained from him. The plasma was obtained by centrifuging the blood sample and frozen until the anticoagulant activities determination.

### 2.2. Sulfated Polysaccharides Isolated from Cloned* Grateloupia filicina*

The milled algal samples were suspended in 40 volumes of H_2_O at room temperature for 2 h, then homogenized, and refluxed at 90°C for 4 h. After cooling to the room temperature, the supernatant was obtained by centrifugation at 5000 rpm for 5 min, concentrated under reduced pressure, and dialyzed in a cellulose membrane against flowing distilled water for 72 h. The dialyzed retention was concentrated under reduced pressure, precipitated by 4 volumes of 95% (V:V) ethanol, and dried. The dried sediment (GSP) was milled and kept for use.

### 2.3. Analytical Methods

Total sugar content was determined using the phenol-sulfuric acid method with glucose as the standard [13]. Sulfate content was determined by barium chloride-gelatin method [14].

Molecular weight of the sample was determined by HPLC Agilent 1260 gel permeation chromatography (GPC) (Agilent Technologies, USA) at 35°C, where ultrapure water was used as mobile phase with a flow rate of 0.5 mL/min. TSK G3000-PWXL column (300 mm × 7.8 mm) and 2140 refractive index detector were used. A series of different molecular weight dextrans purchased from Sigma (St. Louis, Missouri, USA) were used as standards.

The molar ration of monosaccharide composition was determined following Zhang et al. [15]. Generally speaking, a solution of sample (10 mg/mL) was hydrolyzed in 2 M trifluoroacetic acid in a 10 mL ampoule. The ampoule was sealed in a nitrogen atmosphere and hydrolyzed for 4 h at 110°C. Then, the hydrolyzed mixture was neutralized to pH 7 with sodium hydroxide. Later the mixture was converted into its 1-phenyl-3-methyl-5-pyrazolone derivatives and separated by HPLC chromatography. Uronic acid was analyzed by a modified carbazole method [16, 17].

FT-IR spectra were recorded with KBr pellets on a Nicolet FT-IR 360 spectrophotometer. The scan region was 400–4000 cm^−1^ (36 scans, at resolution of 6 cm^−1^).

### 2.4. Anticoagulant Activity Assays

#### 2.4.1. Anticoagulant Action Measured Using Activated Partial Thromboplastin Time (APTT)

APTT was determined using the method of Anderson [18]. In these assays, platelet-poor plasma samples (0.1 mL) were mixed with different amounts of different concentration of the sulfated polysaccharides (from the cloned* Grateloupia filicina*) in 0.9% NaCl (0.05 mL) solution and warmed for 60 s at 37°C. Subsequently, 0.1 mL prewarmed APTT reagent was added and the mixture was allowed to incubate for 5 min at 37°C. Prewarmed 0.25 mol/L calcium chloride (0.1 mL) was then added, and the APTT was determined by semiautomatic blood coagulation analyzer (HF6000-4, Jinan Han Fang Medical Instrument Ltd., China). Solutions of 0.9% NaCl and heparin were used as negative and positive controls, respectively.

#### 2.4.2. Anticoagulant Action Measured Using Prothrombin Time (PT)

PT was determined according to the method of Quick [19]. The reaction mixture containing different concentration samples was incubated with 0.1 mL plasma for 3 min at 37°C, then prewarmed PT reagent was added, and the time for clot formation was determined by semiautomatic blood coagulation analyzer (HF6000-4, Jinan Han Fang Medical Instrument Ltd., China). Solutions of 0.9% NaCl and heparin were used as negative and positive controls, respectively.

#### 2.4.3. Anticoagulant Action Measured Using Thrombin Time (TT)

TT was determined using the method of Denson and Wang [20, 21]. Plasma samples (0.15 mL) were mixed with different concentration samples in 0.9% NaCl (0.05 mL) solution. Subsequently, 0.15 mL of prewarmed TT reagent was added and the time for clot formation was determined by semiautomatic blood coagulation analyzer (HF6000-4, Jinan Han Fang Medical Instrument Ltd., China). Solutions of 0.9% NaCl and heparin were used as negative and positive controls, respectively.

## 3. Results and Discussion

### 3.1. Chemical Analysis

The yield and the chemical compositions of the sample are given in Table 1. From the table, the yield of the sulfated polysaccharides from the cloned* Grateloupia filicina* was 15.75%. The total sugar and the sulfate group content were 40.90% and 19.89%, respectively. The above results were close to the result of Wang [21]. The molecular weight was 11.7 KDa, which was much lower than that of Wang (3.5 × 10^5^ Da) [21] and the result of Athukorala (1357 KDa) [22], which was possible due to the difference of the extraction temperature and time that was effective to the molecular weight.

In this study, neutral monosaccharide constitutions of GSP were analyzed by HPLC. Results showed that galactose was the main sugar forms in the sample which was composed of a small amount of mannose, glucose, xylose, fucose, and glucuronic acid. As shown in Table 1, GSP was high galactose-containing sulfated polysaccharides.

### 3.2. FT-IR Analysis

**Figure 2:**
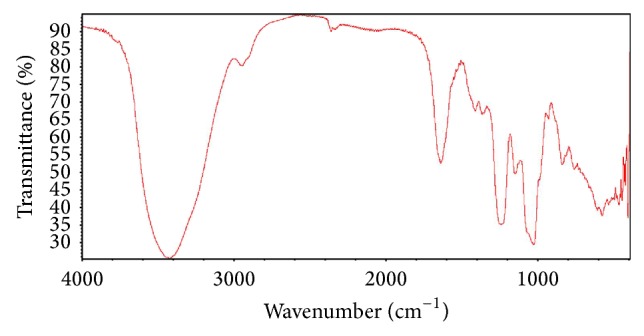
IR spectra of GSP (sulfated polysaccharides from cloned* Grateloupia filicina*).

The FT-IR spectrum of GSP was shown in Figure 2. Typical signals of polysaccharide at about 3423 cm^−1^, 2934 cm^−1^, 1641 cm^−1^, 1408 cm^−1^, 1241 cm^−1^, and 1031 cm^−1^ were clear for the sample. They correspond to the O-H stretching vibrations and the C-H stretching vibrations, respectively. The peaks of 1641 cm^−1^ and 1408 cm^−1^ were corresponding to the carbonyl C=O antisymmetric and symmetric vibrations in uronic acid in the form of salts. 1241 cm^−1^ was corresponding to the S=O asymmetric stretching vibration of sulfate group and 1031 cm^−1^ corresponding to the C-O-H in glucosidal bond or C-O-C stretching vibrations in ring. In addition, the sample showed a band at 845 cm^−1^ indicating a symmetrical C-O-S vibration. These results were agreeable with Wang et al. [23].

### 3.3. Anticoagulant Activity Assays

**Figure 3:**
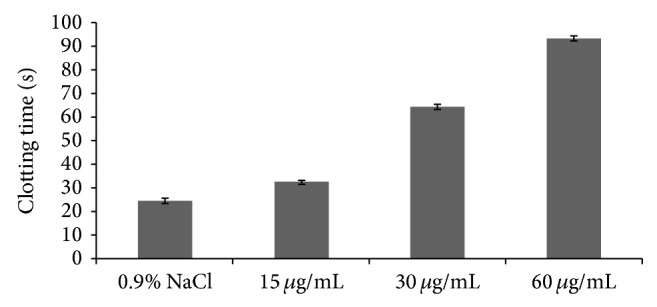
Anticoagulant activity of GSP measured by APTT assay.

**Figure 4:**
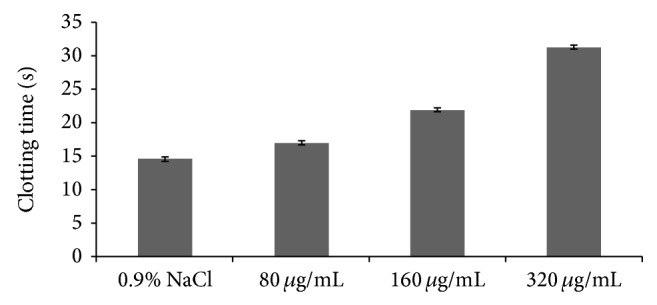
Anticoagulant activity of GSP measured by TT assay.

**Figure 5:**
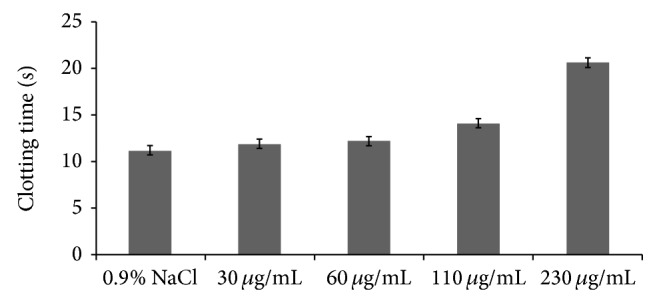
Anticoagulant activity of GSP measured by PT assay.

The anticoagulant activities of GSP were determined by activated APTT, TT, and PT assays* in vitro* that characterize different stages of the coagulation process. The results were listed in Figures 3–5 which showed that APTT, TT, and PT of GSP were prolonged. However, the effect of GSP on clotting time for APTT, TT, and PT was different. It was more obvious for APTT and TT than for PT. From Figure 3, the clotting time was obviously prolonged when the concentration was 15 ug/mL (32.4 s). The clotting time of 0.9% NaCl was 24.5 s, and the clotting time of heparin (3.3 ug/mL) was 40.8 s. From Figure 4, compared with the 0.9% NaCl (14.5 s), when the GSP concentration was 80 ug/mL, the clotting time was prolonged (17.0 s). And the clotting time of heparin (50 ug/mL) was 26.8 s. From Figure 5, compared with the 0.9% NaCl (11.2 s), when the GSP concentration was 110 ug/mL, the clotting time was prolonged (14.1 s). And the clotting time of heparin (7 ug/mL) was 15.6 s.

From the above results, the sulfated polysaccharides of cloned* Grateloupia filicina* showed good anticoagulant activities, but the activities were weaker than that of heparin. As for the APTT assay, GSP was a potential anticoagulant, and the anticoagulant activity increased with increased concentration of GSP. As for TT and PT assays, GSP had a tendency to prolong the clot plasma time. This was agreeable with the previous reference which reported that the sulfated polysaccharides from seaweeds showed anticoagulant activities mainly by inhibition of the intrinsic coagulation pathway [22].

## 4. Conclusion

In this paper, sulfated polysaccharides (GSP) were isolated from the cloned* Grateloupia filicina* which was cultured in Jiaozhou Bay, Qingdao, China. The chemical composition was determined and the results showed that GSP was sulfated polysaccharides of galactose. And GSP showed similar activities with the other sulfated polysaccharides of other seaweeds such as anticoagulant activity. The experiments for APTT, TT, and PT anticoagulant assays indicated that GSP was a good potential anticoagulant. And the cloned* Grateloupia filicina* will be well used for the exploitation of high-value products.

## Figures and Tables

**Table 1:** Yield and chemical composition of the sample (%w/w of dry weight).

Sample	Yield (%)	Total sugar (%)	Sulfate (%)	Molecular weight (KDa)	Neutral sugar (mole ratio)^b^					
					Man	Glc	Gal	Xyl	Fuc	GlucA
GSP^a^	15.75	40.90	19.89	11.7	0.01	0.08	1.00	0.11	0.11	0.02

^a^Sulfated polysaccharides isolated from cloned *Grateloupia filicina*.

^b^Man: mannose; Glc: glucose; Gal: galactose; Xyl: xylose; Fuc: fucose; GlucA: glucuronic acid.
